# Expanded Geographic Distribution and Clinical Characteristics of *Ehrlichia ewingii* Infections, United States

**DOI:** 10.3201/eid2205.152009

**Published:** 2016-05

**Authors:** Rebecca M. Harris, Brianne A. Couturier, Stephan C. Sample, Katrina S. Coulter, Kathleen K. Casey, Robert Schlaberg

**Affiliations:** Author affiliations: University of Utah School of Medicine, Salt Lake City, Utah, USA (R.M. Harris, R. Schlaberg);; Associated Regional and University Pathologists Institute for Clinical and Experimental Pathology, Salt Lake City (B.A. Couturier, R. Schlaberg);; Memorial Hospital and Healthcare Center, Jasper, Indiana, USA (S.C. Sample);; University of Arkansas for Medical Sciences, Little Rock, Arkansas, USA (K.S. Coulter);; Jersey Shore University Medical Center, Neptune, New Jersey, USA (K.K. Casey)

**Keywords:** Ehrlichiosis, Ehrlichia ewingii, Ehrlichia chaffeensis, bacteria, geographic distribution, zoonoses, ticks, vector-borne infections, United States

## Abstract

Ehrlichiosis is a bacterial zoonosis, spread through the bites of infected ticks, that is most commonly caused in the United States by infection with the bacterium *Ehrlichia chaffeensis*. We retrospectively reviewed samples from an 18-month study of ehrlichiosis in the United States and found that *E. ewingii* was present in 10 (9.2%) of 109 case-patients with ehrlichiosis, a higher rate of infection with this species than had previously been reported. Two patients resided in New Jersey and Indiana, where cases have not been reported. All patients with available case histories recovered. Our study suggests a higher prevalence and wider geographic distribution of *E. ewingii* in the United States than previous reports have indicated.

## Abstract 2

This bacterium should be considered as an etiologic agent of tickborne illness that might be missed by serologic testing.

Ehrlichiosis is a bacterial zoonosis spread through the bites of infected ticks. Three species have been identified as causes of ehrlichiosis within the United States: *Ehrlichia chaffeensis*, *E. ewingii*, and an *E. muris*–like pathogen (*1*). Anaplasmosis is a disease with an overlapping clinical syndrome caused by the closely related organism *Anaplasma phagocytophilum* (*2*). *E. chaffeensis* and *E. ewingii* are spread by *Amblyomma americanum* ticks, which are found widely distributed across the eastern and southeastern United States. In contrast, *A. phagocytophilum* is primarily transmitted by *Ixodes scapularis* ticks in the northeastern and upper Midwest regions. *Ix. scapularis* ticks are the possible vector of the *E. muris*–like pathogen. This organism has been detected in *Ix. scapularis* ticks in Minnesota and Wisconsin, where 69 cases of human infection with the *E. muris*–like pathogen have been reported (*1*,*3*).

*E. chaffeensis* is the most common cause of human ehrlichiosis; 1,518 were cases reported in 2013 mainly in south-central, southeastern, and mid-Atlantic states (*4*). Signs and symptoms for this disease include fever, chills, headache, myalgia, malaise, thrombocytopenia, leukopenia, and increased levels of liver enzymes (*5*). Severe infections can occur (case-fatality rate ≈1%) and are a concern in immunocompromised patients (*6*). *E. ewingii* is a less common human pathogen; it accounted for 31 cases of ehrlichiosis in 2013 (*4*). Clinical disease caused by *E. ewingii* has not yet been well characterized.

*E. ewingii* is prevalent in dogs and white-tailed deer throughout the central and southeastern United States, and reported human infections have increased since the disease became reportable in 2008 (*4*,*7**,**8*). The first human cases of infections were reported in 4 patients in Missouri in 1999, three of whom were immunosuppressed (*9*). *E. ewingii* was subsequently reported in 4 symptomatic patients from Missouri, Oklahoma, and Tennessee, all of whom were co-infected with HIV (*10*). One case of *E. ewingii* infection that was likely acquired through platelet transfusion from an asymptomatic donor with tick exposure has been reported (*11*). Most recently, *E. ewingii* was detected in the peripheral blood and bone marrow of a symptomatic 65-year-old woman from Arkansas (*12*). The most consistent clinical findings in these patients were fever and thrombocytopenia. No deaths have been reported.

*Ehrlichia* spp. are obligate intracellular organisms, and morulae (bacterial clusters within cytoplasmic vacuoles) are visualized on peripheral blood films. However, detection of morulae in monocytes (for *E. chaffeensis*) or granulocytes (for *E. ewingii*) has limited sensitivity (*13*). Serologic testing for *E. chaffeensis* can be performed but is insensitive during the acute phase of illness and has limited specificity (*5*,*9*). Specific serologic testing for *E. ewingii* is not available. The extent of cross-reactivity of serologic tests for *E. chaffeensis* with *E. ewingii* is unclear, but use of serologic testing alone might contribute to underreporting of infection with *E. ewingii*. Thus, diagnosis of infection with *E. ewingii* is reliant on species-specific molecular testing. The purpose of this study was to determine the geographic distribution and clinical characteristics of PCR-confirmed *E. ewingii* infections in the United States.

## Methods

We retrospectively reviewed results of 18 months (May 2013–November 2014) of testing for human ehrlichiosis by real-time PCR. All samples submitted to Associated Regional and University Pathologists Laboratories (Salt Lake City, UT, USA) for *Ehrlichia* and *Anaplasma* species by real-time PCR were included in the analysis. PCR-positivity rates for *Ehrlichia* spp. were calculated on the basis of results for individual patients.

We used a real-time PCR for *Ehrlichia* and *Anaplasma* species that detects *E. chaffeensis, E. muris*–like pathogen, *E. ewingii*, and *E. canis* (without differentiating *E.* *ewingii* and *E. canis*) and *A. phagocytophilum*. In brief, nucleic acids extracted from whole blood specimens by using the Chemagic MSM I Automated Extraction Platform and the Chemagen Blood Extraction Kit (Perkin Elmer, Waltham, MA, USA) were amplified by using primers specific for 16S rRNA gene and *Ehrlichia* and *Anaplasma* species–specific probes for identification (Table 1). The reaction was prepared by using a 5× custom real-time Master Mix and 4 mmol/L MgCl_2_ (Promega, Madison, WI, USA) with the following amplification parameters: 50.0°C for 2 min; denaturation at 95.0°C for 2 min; and 50 cycles at 95.0°C for 5 s, 56.0°C for 20 s, and 76.0°C for 20 s on the Rotor-Gene Q apparatus (QIAGEN, Hilden Germany). Melting curve analysis was performed at 95.0°C for 15 s and then from 45°C through 75°C at 1.0°C/step at 5 s/step with continuous fluorescence acquisition.

**Table 1:** Primers and probes used in real-time PCR for *Ehrlichia* and *Anaplasma* species, United States*

Name	Sequence, 5′ → 3′	Concentration, nmol/L	Species detected
Primer 1	GCATTACTCACCCGTCTGCCACT	250	–
Primer 2	CAAGCCTAACACATGCAAGTCGAACG	1,000	–
Probe 1	MGB-FAM-AGGT†T†ATAA†GCA†ATTGTCC-EDQ	200	*E. chaffenisis*
Probe 2	MGB-(AP642)-AAGCTATA†GGCA†GT†TA†TCC-EDQ	200	*E. muris*–like agent
Probe 3	MGB-(AP593)-GGCTATA†A†ATA†A†TTGT†CCG-EDQ	200	*E. canis*
Probe 4	MGB-(AP593)-CTATTTA†GGA†A†TTGT†T†C-EDQ	200	*E. ewingii*
Probe 5	MGB-(AP525)-AAAGAAT†A†A†TCCGTTCG-EDQ	200	*A. phagocytophilum*

*–, these primers are specific for 5 organisms; MGB, minor groove binder; FAM, 6-carboxyfluorescein; EDQ, Eclipse Dark Quencher (Epoch Biosciences Inc., Bothell, WA, USA). †Use of Super A, T, or G (Epoch Biosciences Inc.).

We retained samples positive for *E. ewingii/E. canis* from the initial tests for further analysis for this study. Nucleic acids were extracted from these residual blood specimens, and another real-time PCR specific for a region within the 16S rRNA gene was performed to differentiate *E. ewingii* from *E. canis*. We contacted healthcare providers for *E. ewingii*–positive patients to collect case histories. The study was approved by the University of Utah Institutional Review Board (no. 76713).

## Results

Of 4,177 patients from 41 states who had samples submitted to Associated Regional and University Pathologists Laboratories for detection of *Ehrlichia* and *Anaplasma* species by real-time PCR during an 18-month study period, 99 (2.4%) were positive for *E. chaffeensis*, 10 (0.2%) for *E. ewingii/E. canis*, and 0 for *E. muris*-like pathogen. A total of 179 (4.3%) patients were positive for *A. phagocytophilium*. Positivity rates were calculated by state (Table 2).

**Table 2:** Results for patients tested for infection with *Ehrlichia* and *Anaplasma* species by real-time PCR, United States

State*	No. tested	No. (%) positive		
		*E. chaffeensis*	*E. canis/ewingii*	*A. phagocytophilum*
Arkansas	3	0 (0)	1 (33.3)	0 (0)
Colorado	3	1 (33.3)	0 (0)	0 (0)
Connecticut	23	0 (0)	0 (0)	3 (13)
Georgia	7	1 (14.2)	0 (0)	0 (0)
Illinois	70	4 (5.7)	0 (0)	0 (0)
Indiana	263	25 (9.5)	3 (1.1)	0 (0)
Iowa	4	1 (25)	0 (0)	0 (0)
Kansas	52	10 (19.2)	0 (0)	0 (0)
Kentucky	26	8 (30.8)	0 (0)	0 (0)
Louisiana	11	1 (9.1)	0 (0)	0 (0)
Maine	375	0 (0)	0 (0)	17 (4.5)
Massachusetts	526	0 (0)	0 (0)	40 (7.6)
Michigan	3	1 (33.3)	0 (0)	0 (0)
Minnesota	930	0 (0)	0 (0)	44 (4.7)
Missouri	94	15 (15.9)	4 (4.3)	0 (0)
Nebraska	69	8 (11.6)	0 (0)	1 (1.4)
New Hampshire	708	1 (0.1)	0 (0)	51 (7.2)
New Jersey	202	7 (3.5)	1 (0.5)	5 (2.5)
New York	283	3 (1.1)	1 (0.4)	6 (2.1)
Pennsylvania	72	2 (2.8)	0 (0)	1 (1.4)
Tennessee	150	6 (4)	0 (0)	0 (0)
Texas	52	2 (3.8)	0 (0)	1 (1.9)
Utah	16	1 (6.3)	0 (0)	0 (0)
Virginia	28	0 (0)	0 (0)	1 (3.6)
Washington	24	2 (8.3)	0 (0)	0 (0)
Wisconsin	68	0 (0)	0 (0)	9 (13.2)

*States for which all patents showed negative results are Alabama (n = 2), Arizona (n = 11), California (n = 19), Florida (n = 9), Hawaii (n = 1), Idaho (n = 1), Montana (n = 1), Nevada (n = 3), North Carolina (n = 6), North Dakota (n = 2), Ohio (n = 25), Oregon (n = 8), Rhode Island (n = 3), South Dakota (n = 21), and Wyoming (n = 3).

All 10 *E. ewingii/E. canis*–positive cases were subsequently identified as *E. ewingii*, accounting for 10 (9.2%) of 109 ehrlichiosis cases during the study period.  *E. ewingii*–positive samples were from 9 men and 1 woman (median age 58 years, range 24–74 years). The samples were from Missouri (n = 4), Indiana (n = 3), Arkansas (n = 1), New Jersey (n = 1), and New York (n = 1) and were collected in June (n = 1), July (n = 3), August (n = 4), and September (n = 2). Case histories and laboratory results were obtained for 5 patients (Table 3).

**Table 3:** Associated laboratory findings for 5 patients infected with *Ehrlichia ewingii*, United States

Laboratory finding	Patient				
	1	2	3	4	5
Minimum leukocyte count, × 10^3^ cells/μL	3.5	4.5	2.7	2.6	2.8
Minimum platelet count, × 10^3^/μL	128	179	102	92	100
Maximum aspartate aminotransferase level, U/L	24	115	38	28	91
Maximum alanine aminotransferase level, U/L	26	279	38	28	78
Maximum alkaline phosphatase level, U/L	77	90	85	87	146

Case-patient 1 was 24-year-old man (landscaper) from New Jersey who had nausea, vomiting, and fevers in August 2014. The patient had no known tick bites and no major underlying concurrent illnesses. At the time of presentation, his temperature was 102°F; results of a physical examination were otherwise unremarkable. Laboratory testing showed thrombocytopenia, and blood cultures obtained at presentation were negative for bacterial growth. Serologic testing for Lyme disease by ELISA showed antibodies against *Borrelia burgdorferi*. Western blot showed a positive result for IgM and a negative result for IgG. Serologic testing for *Ehrlichia* spp. was not performed. The patient received a 2-week course of doxycycline, and his symptoms resolved.

Case-patient 2 was a 38-year-old man from Missouri who had a history of headache and fever for 10 days in August 2014. The patient had no known tick exposure, and his medical history was unremarkable. At the time of presentation, his temperature was 102°F. He had an eczematous rash on his left upper leg and increased levels of liver enzymes. Results of serologic testing for *E. chaffeensis* were negative. The patient received a 1-week course of doxycycline, and his symptoms resolved.

Case-patient 3 was a 73-year-old man from Indiana who had subjective fevers, mild left lower abdominal pain, myalgia, and malaise. The patient reported a tick bite on his abdomen 2–3 weeks before presentation. His medical history included type 1 diabetes, hypertension, and stroke. Results of a physical examination were unremarkable. He was afebrile at presentation but had leukopenia and thrombocytopenia. Results of serologic testing for Lyme disease (total antibodies against *B. burgdorferi* by ELISA) were negative. Serologic testing for *Ehrlichia* spp. was not performed. The patient received a 10-day course of doxycycline, and his symptoms resolved.

Case-patient 4 was a 74-year-old man from Arkansas who had malaise, arthralgia, fever, and nonbloody diarrhea in for 2 weeks in July 2014. The patient reported 2 tick bites 2 weeks before presentation. His medical history included diffuse large B-cell lymphoma (treated with gemcitabine), lung cancer (treated with a right lobectomy), chronic obstructive pulmonary disease that required supplemental oxygen, coronary artery disease, and hypothyroidism. At presentation, he had hypotension (79/46 mm Hg), tachycardia (142 beats/min), and a fever (temperature 100.1°F). Laboratory testing showed leukopenia, thrombocytopenia, and morulae in neutrophils on a peripheral blood smear. Results of serologic testing for *E. chaffeensis* were negative. The patient received a 3-week course of doxycycline and showed clinical improvement; however, he died 1 month later of unrelated bacterial sepsis.

Case-patient 5 was a 73-year-old man from Missouri who had headache, fever, and nausea in August 2014. The patient reported a tick bite ≈2 months before presentation. His medical history included bladder cancer (treated with immunotherapy), a melanoma on his right arm (treated with surgical excision), type 1 diabetes, and hypothyroidism. He had leukopenia, thrombocytopenia, and increased levels of liver enzymes. Blood cultures at presentation and serologic results for *E. chaffeensis* were negative. The patient received a 10-day course of doxycycline, and his symptoms resolved.

## Discussion

In our study, *E. ewingii* accounted for 10 (9.2%) of 109 cases of *Ehrlichia* spp. infections, compared with only 31 (2.0%) of 1,518 reported human ehrlichiosis cases in the United States (*4*). Underreporting might be caused by successful empirical treatment without etiologic diagnosis; missed *E. ewingii* infections by serologic tests for *E. chaffeensis* in the acute phase of illness (case-patients 2, 4, and 5); and limited availability of molecular tests. Even with molecular testing, cases might be missed because of non-optimal timing of specimen collection and limited sensitivity of the assay, such as with testing after resolution of bacteremia.

Human infections with *E. ewingii* naturally acquired have been reported in Arkansas, Missouri, Oklahoma, and Tennessee (*9**,**10**,**12*). We now describe cases of infection in Indiana and New Jersey. The location of these cases is consistent with the known range of the vector (*A. americanum* ticks). *E. ewingii* has previously been reported in *A. americanum* ticks collected in New Jersey (*14*).

The 5 case-patients reported in this study had symptoms of classical ehrlichiosis, including fever, myalgia, malaise, and headache. Thrombocytopenia and leukopenia were the most consistent associated laboratory findings. All patients improved after treatment with doxycycline treatment, despite in some instances, major underlying disease. *E. ewingii* should be considered as an etiologic agent of tickborne febrile illness in the central and eastern United States that may be missed by serologic testing.
